# Psychometric Modelling of Longitudinal Genetically Informative Twin Data

**DOI:** 10.3389/fgene.2019.00837

**Published:** 2019-10-16

**Authors:** Inga Schwabe, Zhengguo Gu, Jesper Tijmstra, Pete Hatemi, Steffi Pohl

**Affiliations:** ^1^Methodology and Statistics, Tilburg University, Tilburg, Netherlands; ^2^Translational Neurogenomics, QIMR Berghofer Medical Research Institute, Brisbane, QLD, Australia; ^3^Political Science, Pennsylvania State University, University Park, Pennsylvania, United States; ^4^Methods and Evaluation/Quality Assurance, Freie Universität Berlin, Berlin, Germany

**Keywords:** genetic correlations, item response theory, longitudinal data, measurement error, phenotypic stability, psychometrics, sum–scores, twin study

## Abstract

The often-used A(C)E model that decomposes phenotypic variance into parts due to additive genetic and environmental influences can be extended to a longitudinal model when the trait has been assessed at multiple occasions. This enables inference about the nature (e.g., genetic or environmental) of the covariance among the different measurement points. In the case that the measurement of the phenotype relies on self-report data (e.g., questionnaire data), often, aggregated scores (e.g., sum–scores) are used as a proxy for the phenotype. However, earlier research based on the univariate ACE model that concerns a single measurement occasion has shown that this can lead to an underestimation of heritability and that instead, one should prefer to model the raw item data by integrating an explicit measurement model into the analysis. This has, however, not been translated to the more complex longitudinal case. In this paper, we first present a latent state twin A(C)E model that combines the genetic twin model with an item response theory (IRT) model as well as its specification in a Bayesian framework. Two simulation studies were conducted to investigate 1) how large the bias is when sum–scores are used in the longitudinal A(C)E model and 2) if using the latent twin model can overcome the potential bias. Results of the first simulation study (e.g., AE model) demonstrated that using a sum–score approach leads to underestimated heritability estimates and biased covariance estimates. Surprisingly, the IRT approach also lead to bias, but to a much lesser degree. The amount of bias increased in the second simulation study (e.g., ACE model) under both frameworks, with the IRT approach still being the less biased approach. Since the bias was less severe under the IRT approach than under the sum–score approach and due to other advantages of latent variable modelling, we still advise researcher to adopt the IRT approach. We further illustrate differences between the traditional sum–score approach and the latent state twin A(C)E model by analyzing data of a two-wave twin study, consisting of the answers of 8,016 twins on a scale developed to measure social attitudes related to conservatism.

## Introduction

Even in the genomics era, twin studies remain useful to estimate the relative importance of genetic and environmental influences, in particular when the power of more complex models is restricted through the low amount of phenotypic variance explained in genome-wide association studies (GWAs) that use direct genetic information from genotyped individuals. In the commonly used ACE model, the total phenotypic variance is decomposed into components due to additive genetic (A) influences, common-environmental (C) influences that are shared by both family members and unique-environmental (E) influences that are unique to the individual twin (Jinks and Fulker, 1970).

When the trait has been assessed at multiple occasions, this model can be extended to a longitudinal design to analyze the nature of the variance at all time points as well as the covariance among them (Boomsma et al., 2002). Potentially, this makes results very informative, as it enables us not only to investigate phenotypic covariance, but we can also decompose this covariance further into genetic and environmental components. Imagine for example that we have measured the mathematical ability of monozygotic (MZ) and dizygotic (DZ) twin pairs at four different time points (e.g., at the age of 5, 10, 15, and 20). As in the univariate ACE model, we can determine the relative importance of genetic and environmental influences in creating differences in the phenotype. Additionally, we can also quantify the importance of both components among the different time points. For example, a hypothesis could be that in earlier years (e.g., age 5 to 10) mainly genetic influences are important while in later years environmental influences become more dominant as secondary schools on different educational levels create more environmental variance. Secondly, the data allows us to determine phenotypic stability: How stable is the twins’ performance among the different time points? We can do this by simply calculating the covariance among the time points. While the latter is a property of every longitudinal design, the particular strength of the longitudinal *twin* design is that we can further decompose this covariance into genetic and environmental components. In other words, what is the relative importance of either genetic or environmental influences in explaining this covariance? In addition to providing the means to answer these substantive questions, longitudinal measures can also increase the statistical power to detect genetic and/or environmental effects (Schmitz et al., 1998).

The longitudinal design has been used extensively in the field of behavior genetics, leading to a number of relevant findings. For example, Long et al. (2017) used a Swedish population-based sample consisting of male twin pairs (1,532 MZ and 1,940 DZ twin pairs) and 66,033 full male sibling pairs born less than 2 years apart where alcohol use disorder (AUD) was assessed during three age periods (18–25, 26–33, and 33–41). They found that, although the heritability of AUD seemed to be stable over time, there were two major genetic factors that contributed to AUD risk—one beginning at ages 18–25 with a modest decline in importance over time and one of less impact beginning at ages 26–33 with a modest increase in importance by ages 33–41. Nivard et al. (2015) conducted a longitudinal study on symptoms of anxiety and depression (SxAnxDep) across the lifespan. They combined data from 49,524 twins where SxAnxDep were assessed repeatedly with a maximum of eight assessments over a 25-years period. Using the genetic simplex model [for more details, see Boomsma and Molenaar (1987)], they found that the substantial phenotypic stability in SxAnxDep could be explained mainly by genetic effects. Furthermore, environmental influences contributed to change (e.g., their importance increased with age), but also to short-term stability.

Aforementioned studies exemplify how the results of longitudinal twin studies contribute to our understanding of the etiological mechanisms underlying a trait by elucidating the nature of genetic and environmental influences over time. For example, the finding by Nivard et al. (2015) that environmental effects contributed to change but also to short-term stability suggests that, in clinical practice, an improvement in SxAnxDep can be accomplished by positive environmental experiences, such as beneficial therapy or positive life events. Contrarily, increases of SxAnxDep can be caused by negative experiences, such as adverse life events. These implications underline the importance of addressing the environment in therapy (e.g. increasing social support or involving significant others). Additionally, their results have several implications for future research. For example, the fact that they have observed little age-related heterogeneity in genetic effects implies that gene-finding studies should strive for big samples that may include adults aged between 18 and 63 instead of stratifying samples in age groups. Overall, the findings of longitudinal twin studies 1) have important implications for practice, and 2) can help to formulate recommendations for subsequent research. Arguably, when a longitudinal twin analysis finds that different genetic factors are at play in explaining individual differences in a certain trait for different age groups, this suggests that a genome-wide association study (GWAS) on that same trait should strive for big samples of certain age groups to avoid hampering of gene-findings through an increased amount in genetic heterogeneity.

### Potential Issues of Sum–Scores

As is common across all behavior sciences, in the field of behavior genetics, often, self-reported measures consisting of multiple items (e.g., a mathematical ability test or a personality questionnaire) are used to measure the phenotype of interest. Traditionally, the scores obtained on the multiple items are aggregated into a sum–score by adding up an individual twin’s answers to all items in a questionnaire or test and then used as a proxy for the latent variable of interest (e.g., cognitive ability or personality trait). However, using this approach comes with a number of disadvantages. First, and most importantly, the uncertainty (e.g., the measurement error) that results from not observing the latent trait directly is usually ignored when the sum–score approach is used. This results in a confounded measure of the phenotype and impacts estimated variances and relationships with other variables (Fox and Glas, 2003; van den Berg et al., 2007). Another disadvantage is that the sum–score approach is not very flexible when it comes to the handling of missing values. Twins might for example not answer all questions on a questionnaire but skip items on sensitive topics (e.g., questions related to socially undesirable behavior) or not reach the end of a test due to time limits. Although more complex methods are available, in that case, often values are imputed (e.g., the respondent’s mean response on all available items) but the uncertainty of the imputed values is not taken into account as standard errors and confidence intervals are calculated as if there were no missing item scores. Furthermore, imputation of the respondent’s mean response on the available item responses does not take the item characteristics (such as item difficulty) into account, which will be a problem if responses are not missing completely at random. Lastly, foundational to the interpretation of any longitudinal design is that the assumption of measurement invariance is met. This means that the same trait is measured in the same way across the different time points (Millsap, 2011). The absence of measurement invariance, however, cannot be assessed or corrected for when the sum–score approach is used (Millsap, 2010). Lubke et al. (2012) showed that, if undetected, absence of measurement invariance will be confounded with group differences in heritabilities and consequently can lead to bias, such as the spurious finding of genotype by environment interaction or (the absence of) scalar sex-limitations. Using analytic derivations, simulation studies, and an empirical analysis of aggression in children aged 3–12 years, Luningham et al. (2017) investigated to which extent violations of measurement invariance lead to bias in the results of a latent growth model fitted to sum–scores. The results showed that measurement non-invariance across age can lead to bias in estimates of the growth mean and variances. However, results also suggested that the results of a genetic variance decomposition of growth factors is not biased due to measurement non-invariance across age, provided that there is no measurement non-invariance across birth-order and zygosity in twins. Another potential issue of longitudinal designs is that measurement instruments often change, for instance because certain questionnaire items might be less suitable for an older age group. For example, in intelligence research, often standardized scores are used based on different versions of a test that is tailored at different age groups (e.g., different versions of the commonly used Raven Progressive Matrices test, Raven, 2000). Such changes in assessment indicators hamper the use of sum–scores for evaluation of change.

Motivated by the fact that this source of potential bias is the most relevant when estimating heritability, in this paper, we concentrate on one particular limitation of the sum–score approach: not taking into account measurement error. Particularly when a scale is used with only a few items and unreliability is not taken into account, estimated correlations between measures related to the phenotype will be suppressed—a phenomenon that is referred to as attenuation in the statistical literature. In the cross-sectional case (e.g., when only a single measurement is considered), van den Berg et al. (2007) showed that attenuation leads to an underestimation of heritability, particularly when the sum–score distribution is skewed. When decomposing the raw sum–scores aggregated over 14 dichotomous items (either with or without log transformation) under an AE model, the heritability point estimate dropped from the true value of 72% to an estimate of approximately 42%. They furthermore show that this effect depends on 1) the number of items (e.g., this bias was the most severe when sum–scores were based on a total of five items but vanished when answers to 100 items were simulated) and 2) the true correlation among MZ and DZ twins (e.g., with five items, a true correlation of 0.90 was attenuated to a correlation of 0.55 and a true correlation of 0.10 was attenuated to a correlation of 0.06).

### Item Response Theory—A Solution to the Problem?

Alternatively to calculating sum–scores, we can also apply latent variable modelling, a framework that directly accounts for measurement error. There are different latent variable modelling traditions (e.g., structural equation modelling and item response theory). As in psychological assessments measurements are often not metric, but categorical or ordinal, we here focus on models of the item response theory (IRT) framework.

For illustrative purposes, we focus on a simple model for explaining the idea. Note however that the argumentation also applies to other latent variable models. For dichotomous responses (e.g., when responses are scored as 1 = correct and 0 = incorrect), the most basic IRT model is the Rasch model (Rasch, 1960). In the Rasch model, the probability of a correct answer to item *k* (e.g., of a mathematics test) by twin *j* from family *i*, *P*(*Y* *_ijk_* = 1), is modeled as a logistic function of the difference between the twin’s latent trait score (e.g., mathematical ability) and the difficulty of the item:

(1) $$P\left(Y_{ijk}=1\right)=\frac{exp\left(\theta_{ij}-b_{k}\right)}{1+exp\left(\theta_{ij}-b_{k}\right)}$$

where θ*_ij_* refers to the latent trait score of individual twin *j* from family *i*. *b* *_k_* denotes the difficulty of item *k* which represents the trait level associated with a 50% chance of endorsing an item. An underlying assumption of the Rasch model is that each item is equally informative for the latent trait (e.g., comparable to equal factor-loadings in structural equation modelling).

van den Berg et al. (2007) showed that potential bias due to an attenuation can be solved by applying an IRT model within the genetic model. Crucial to this approach is that both, genetic and IRT models, are estimated *simultaneously* such that the estimation takes into account the unreliability of the measurement (Béguin and Glas, 2001). This is computationally challenging, as multiple integrals have to be solved due to the multilevel nature of the twin design [estimating between and within twin pair (co-)variance]. Alternatively, the Bayesian framework with Markov chain Monte Carlo (MCMC) algorithms can be used to estimate the model (see also van den Berg et al., 2006; van den Berg et al., 2007).

### Research Questions

For the case where a single measurement occasion is considered, van den Berg et al. (2007) showed how severe the bias of using sum–scores can be as well as the utility of IRT modelling within the genetic model for solving this issue. As far as we know, the impact of measurement error and the utility of latent variable modelling for longitudinal genetic data, in which covariances are further decomposed into genetic- and environmental sources, has not been shown. In fact, so far, behavior genetics researchers commonly use sum–scores for longitudinal analyses. However, the issue of bias due to attenuation can be expected to be especially problematic for longitudinal twin studies, as there the relationship between many more latent variables is considered: in addition to the correlations that are studied *within* each time point, these studies also consider the association between latent variables *across* time points. Each of these correlations can be expected to suffer from attenuation, which may create a misleading picture of both the heritability and the stability of traits. As such, the impact of the measurement error might even be more severe than in the cross-sectional case.

In this paper, we first present a latent state longitudinal A(C)E model that integrates IRT modelling into longitudinal twin modelling and then the results of two simulation studies to answer the following research questions:

1. How large is the bias when the sum–score approach is used in the longitudinal case?
2. Can we reduce the bias by using the latent state longitudinal model?

In both simulation studies, we simulated item-level longitudinal twin data which we then analyzed using both the traditional sum–score approach and the latent state longitudinal A(C)E model introduced here. The first simulation study was conducted to get an overview of how the classical approach (e.g., using sum–scores) and the psychometric approach (e.g., analyzing item-level data) compare in terms of bias. For an easier interpretation and to be able to investigate a large number of scenarios, a simple AE decomposition model was used where the variance–covariance matrix is only decomposed into additive genetic- and unique-environmental influences. While the magnitude of variance explained by additive genetic- and unique-environmental influences was fixed, we manipulated the magnitude of covariance due to these two sources. Based on the results of this study, the worst case scenario (e.g., the combination of covariance among additive genetic- and environmental influences at both time points that led to the largest bias) was selected and, under this scenario, an ACE model was used for the data generation where we manipulated the magnitude of covariance due to common-environmental influences.

To further illustrate the difference between the traditional sum–score approach and the proposed psychometric approach, we analyzed data from a two-wave twin study, consisting of the answers of 8,016 twin pairs at two different time points on a scale developed to measure attitudes related to conservatism.

### Latent State Longitudinal A(C)E Twin Model

A broad range of different longitudinal models exist in the field of behavior genetics. Some of the longitudinal models typically used in psychometric research follow the same parametrization (without the genetic modelling) but are known in psychometrics under a different name (e.g., simplex genetic models and auto regressive models), while others have not been applied to genetic modelling yet (e.g., latent change models). These models all differ in their substantive interpretations and an overview of the existing models in both genetics and psychometrics is beyond the scope of this paper. In this paper, we propose the latent state longitudinal A(C)E twin model, where we combine item response modelling with longitudinal twin modelling. In the psychometric part of this model (the latent state model, Steyer et al., 1992), an IRT model is applied to model raw item-level data at each time point. The genetic part of this model is known as the independent pathway model in the twin literature (see e.g. Neale and Cardon, 1992) and consists of decomposing the phenotypic variance into additive genetic (A), common-environmental (C) and unique-environmental (E) influences at each time point separately, such that we can estimate stability of these components across time.

For the sake of simplicity, we will use a model with only two time points throughout the paper, but note that an extension to multiple time points is straightforward. To estimate the latent state longitudinal A(C)E twin model, we adopt the Bayesian framework and use off-the-shelf MCMC methods. In the following, we will present the model for MZ and DZ twins separately. The latent variables (A, C and E) in the models are all modeled here as multivariate normal distributed variables (e.g., correlated among the two time points), but note that this presentation is not standard notation, but serves the Bayesian estimation procedure where the model specification consists of conditional distributions.

#### Monozygotic Twins

We assumed a multivariate normal distributed additive genetic effect for both considered time points. For every MZ family *i*, we then have:

(2) $$\begin{array}{c} A1_{i}\\ A2_{i} \end{array}∼\mathrm{MVN}\left(\left(\begin{array}{c} \mu_{A1}\\ \mu_{A2} \end{array}\right)\;,\;\Sigma_{A}\right)\;$$

where *A*1*_i_* and *A*2*_i_* represent additive genetic influences for family *i* at the first and second time point, respectively. An assumption of the genetic model is that both, *A*1 and *A*2, have an expected value of zero, e.g. μ_A1_ = μ*_A_*_2_ = 0 [see for example Fisher (1918) or Jinks and Fulker (1970)]. Σ***_A_*** denotes the variance–covariance matrix of genetic influences at both time points and is equal to:

(3) $$\Sigma_{A}=\left[\begin{array}{cc} \sigma_{A1}^{2} & \sigma_{AC}^{2}\\ \sigma_{AC}^{2} & \sigma_{A2}^{2} \end{array}\right]$$

where $\sigma_{A1}^{2}$ and $\sigma_{A2}^{2}$ represent variance (at the first and second time point respectively) that can be explained by additive genetic influences. $\sigma_{AC}^{2}$ denotes covariance among the two time points that can be explained by additive genetic influences.

Then, common-environmental effects for every family *i* at both time points, that is *C*1*_i_* and *C*2*_i_*, were assumed to be multivariate normal distributed:

(4) $$\begin{array}{c} C1_{i}\\ C2_{i} \end{array}∼\mathrm{MVN}\left(\left(\begin{array}{c} \mu_{C1}\\ \mu_{C2} \end{array}\right)\;,\;\Sigma_{C}\right)\;$$

where, as for additive genetic influences, we assumed that expected values are equal to zero, that is μ_C1_ = μ_C2_ = 0 and Σ***_C_*** denotes the variance–covariance matrix of common-environmental influences at both time points, which is equal to:

(5) $$\Sigma_{C}=\left[\begin{array}{cc} \sigma_{C1}^{2} & \sigma_{CC}^{2}\\ \sigma_{CC}^{2} & \sigma_{C2}^{2} \end{array}\right]$$

where $\sigma_{C1}^{2}$ and $\sigma_{C2}^{2}$ represent variance (at the first and second time point respectively) that can be explained by common-environmental influences and $\sigma_{CC}^{2}$ denotes covariance among the time points that can be explained by common-environmental influences.

We then modeled the phenotypes θ*_ijt_* of every twin *j* from family *i* at time point *t* = {1,2} using two multivariate normal distributions:

(6) $$\text{Twin }1:\;\begin{array}{c} \theta_{i11}\\ \theta_{i12} \end{array}∼MVN\left(\left(\begin{array}{c} \mu_{1}+C1_{i}+A1_{i}\\ \mu_{2}+C2_{i}+A2_{i} \end{array}\right)\;,\;\Sigma_{E}\right)\;$$

(7) $$\text{ Twin }2:\;\begin{array}{c} \theta_{i21}\\ \theta_{i22} \end{array}∼MVN\left(\left(\begin{array}{c} \mu_{1}+C1_{i}+A1_{i}\\ \mu_{2}+C2_{i}+A2_{i} \end{array}\right)\;,\;\Sigma_{E}\right)\;$$

where θ*_i_*_11_ and θ*_i_*_12_ represent the phenotypic trait of twin 1 of family *i* at time point 1 and 2 respectively, μ_1_ and μ_2_ are the phenotypic means at time point 1 and 2 respectively (where μ_1_ is fixed to zero to identify the IRT model) and Σ***_E_*** is the variance–covariance matrix of unique-environmental influences at both time points, which is equal to:

(8) $$\Sigma_{E}=\left[\begin{array}{cc} \sigma_{\text{E}1}^{2} & \sigma_{\text{EC}}^{2}\\ \sigma_{\text{EC}}^{2} & \sigma_{\text{E}2}^{2} \end{array}\right]$$

where $\sigma_{E1}^{2}$ and $\sigma_{E2}^{2}$ represent residual variances at time point 1 and 2 respectively and $\sigma_{EC}^{2}$ denotes covariance that may be due to environmental influences or due to measurement error. Note that without a measurement model, we cannot distinguish between these two sources of variance. However, if the latent phenotype is modeled as a latent variable (e.g. using an IRT model), measurement error can be accounted for and $\sigma_{E1}^{2}$ and $\sigma_{E2}^{2}$ only capture variance due to unique-environmental influences.

Simultaneous to the decomposition model above, for twin *j* from family *i* at time point *t*, the latent phenotype was modeled based on the one parameter logistic IRT model:

(9) $$\text{ln}\left(P_{ijtk}/\left(1-P_{ijtk}\right)\right)=\theta_{ijt}-\beta_{k}$$

where the probability for endorsing an item *k* at time point *t* is modeled as a function of the difference between a twin’s latent trait score θ at time point *t* and the item difficulty parameter β*_k_*. In both simulation studies and in the empirical data application, we assumed that item difficulty parameters are the same at both time points to meet the assumption of measurement invariance (Meredith, 1993; Borsboom, 2008). Note however that one needs to check whether this assumption actually holds when applying the model to empirical data.

#### Dyzygotic Twins

The model is essentially the same for non-identical twins, but while the total genetic variance is assumed to be the same for DZ and MZ twins, the genetic covariance in MZ twins is twice as large as in DZ twins, as DZ twin pairs share on average only 50% of their polymorphic alleles (e.g., the same level of genetic similarity as found in non-twin siblings). To model a genetic correlation of $\frac{1}{2}$ for DZ twins, we first modeled a multivariate normal distributed additive genetic effect for both considered time points that is the *same* for every DZ twin family and then a multivariate normal distributed additive genetic effect that is *unique* for every individual twin *j*. The common additive genetic effect at both time points, *aDZ*1*_i_* and *aDZ*2*_i_* were modeled as follows:

(10) $$\begin{array}{c} aDZ1_{i}\\ aDZ2_{i} \end{array}∼MVN\left(\left(\begin{array}{c} \mu_{A1}\\ \mu_{A2} \end{array}\right)\;,\;\frac{1}{2}\Sigma_{A}\right)\;$$

where, as for MZ twins, expected values of genetic influences at both time points, *aDZ*1 and *aDZ*2, were assumed to be equal to zero (e.g., μ*_A_*_1_ = μ*_A_*_2_ = 0) and the variance–covariance matrix, Σ***_A_*** is the same as for MZ twins (see Equation 3). At both time points, a unique additive genetic effect for every individual twin *j* from family *i* was modeled, with expected value equal to a family’s common additive genetic effect:

(11) $$\begin{array}{c} ADZ1_{ij}\\ ADZ2_{ij} \end{array}∼MVN\left(\left(\begin{array}{c} aDZ1_{i}\\ aDZ2_{i} \end{array}\right)\;,\;\frac{1}{2}\Sigma_{A}\right)\;$$

As for MZ twins, for every family *i*, multivariate normal distributed common-environmental effects at both time points were modeled (see Equation 4). Similar to the model for MZ twins, an ACE decomposition on the latent variable then yields:

(12) $$\text{Twin }1:\;\begin{array}{c} \theta_{i11}\\ \theta_{i12} \end{array}∼MVN\left(\left(\begin{array}{c} \mu_{1}+ADZ1_{i1}+C1_{i}\\ \mu_{2}+ADZ2_{i1}+C2_{i} \end{array}\right)\;,\;\Sigma_{E}\right)\;$$

(13) $$\text{Twin }2:\;\begin{array}{c} \theta_{i21}\\ \theta_{i22} \end{array}∼MVN\left(\left(\begin{array}{c} \mu_{1}+ADZ1_{i1}+C1_{i}\\ \mu_{2}+ADZ2_{i1}+C2_{i} \end{array}\right)\;,\;\Sigma_{E}\right)\;$$

where the variance–covariance matrix, Σ***_E_***, was the same as for MZ twins (see Equation 8). As for MZ twins, a Rasch IRT model for the item responses was assumed for every twin *j* from family *i* at time point *t* (see Equation 9).

#### Parameters of Interest

Under this model, narrow-sense heritability *h* ^2^ can be estimated separately for both time points (e.g., $h_{T1}^{2}$ for the first time point and $h_{T2}^{2}$ for the second time point), defined as the proportion of the total phenotypic variance at the respective time point that can be explained by additive genetic variance:

(14) $$h_{T1}^{2}=\frac{\sigma_{A1}^{2}}{\sigma_{P1}^{2}}=\frac{\sigma_{A1}^{2}}{\sigma_{A1}^{2}+\sigma_{C1}^{2}+\sigma_{E1}^{2}}$$

(15) $$h_{T2}^{2}=\frac{\sigma_{A2}^{2}}{\sigma_{P2}^{2}}=\frac{\sigma_{A2}^{2}}{\sigma_{A2}^{2}+\sigma_{C2}^{2}+\sigma_{E2}^{2}}$$

Furthermore, we can estimate the correlation between the latent variables (A, C, and E) at both time points:

(16) $$\rho \left(A1,A2\right)=\frac{\sigma_{AC}^{2}}{\sqrt{\sigma_{A1}^{2}\sigma_{A2}^{2}}}$$

(17) $$\rho \left(C1,C2\right)=\frac{\sigma_{CC}^{2}}{\sqrt{\sigma_{C1}^{2}\sigma_{C2}^{2}}}$$

(18) $$\rho \left(E1,E2\right)=\frac{\sigma_{EC}^{2}}{\sqrt{\sigma_{E1}^{2}\sigma_{E2}^{2}}}$$

where ρ(*A*1, *A*2) denotes the correlation between additive genetic influences at the first and second time point, ρ(*C*1, *C*2) depicts the correlation between common-environmental influences at the two time points and ρ(*E*1, *E*2) represents the correlation between unique-environmental influences at the first and second time point.

#### Estimation

van den Berg et al. (2007) showed that, in order to take full advantage of the IRT approach, the genetic twin model and the IRT model have to be fitted within one step. Here, we use Bayesian statistical modelling to estimate all parameters of the latent trait state twin model simultaneously. In the Bayesian framework, statistical inference is based on the joint posterior density of the model parameters, which is proportional to the product of a prior probability and the likelihood function of the data (for further reading see e.g. Box and Tiao, 1972). We use a Markov chain Monte Carlo (MCMC) algorithm called Gibbs sampling (Geman and Geman, 1984; Gelfand and Smith, 1990; Gelman et al., 2004) to obtain this joint posterior density. The algorithm works by iteratively drawing samples from the full conditional distributions of all unobserved parameters of a model. The full conditional distribution relates to the distribution of a parameter given the current values of all other relevant parameters of the model (Gilks et al., 1996). In each iteration of the Gibbs sampling algorithm, a sample is taken from the conditional distribution of every parameter in the model, given the current values of the other relevant parameters of the model. It can be shown that after a number of so-called “burn-in” iterations, subsequent draws can then be seen as draws from the joint posterior distribution of all parameters.

#### Prior Distributions

Using a Bayesian approach, prior distributions have to be defined for all unobserved parameters. Here, we describe the general settings for the latent state longitudinal A(C)E twin model. This means that we chose relativity non-informative prior distributions, resulting in prior distributions that are flat relative to the likelihood function and thus have minimal impact on the posterior distribution. This implies that we do not have any prior guess or knowledge about how the data are generated prior to observing them. Note that, depending on the data at hand, other prior settings might be more reasonable, for example when earlier research has shown that heritability decreases with age.

For the covariance matrices, Σ***_A_***, Σ***_C_***, and Σ***_E_***, we used independent inverse Wishart distributions, Σ^−^ **^1^** ∼ *Wishart*(*L*, *T*), where *L* is a *T* by *T* scale matrix and *k* refers to the degree of freedoms, with *T* being equal to the total number of time points.

In the sum–score approach, two independent normal distributed prior distributions were used for the phenotypic mean at both time points (e.g., μ_1_ ∼ *N*(0, 10) and μ_2_ ∼ *N*(0, 10)). In the IRT analysis, the same prior distribution was used for the second time point, but the phenotypic mean was set to zero at the first time point (e.g., μ_1_ = 0) in order to identify the IRT model. For the difficulty item parameters, we used independent normal distributions as prior distributions [e.g., β*_k_* ∼ *N* (0, 10)].

## Simulation Study 1

Simulation study 1 was conducted to investigate how the classical approach (e.g., using a sum–score) and the psychometric approach (e.g., analyzing item-level data as described above) compare in terms of bias.

As it is generally known that additive genetic influences and common-environmental influences are less well resolvable compared to additive genetic influences and unique-environmental influences or common-environmental influences and unique-environmental influences (see e.g., Martin et al., 1978), for an easier interpretation, a simple AE model was used in the first simulation study. This was also done to gain more computational power in order to be able to investigate a larger number of scenarios. Based on the first simulation study, the worst case scenario (e.g., the one with the largest bias) was selected and in a second simulation study, this scenario was extended to an ACE model for which we manipulated the magnitude of covariance among time points explained by common-environmental influences.

### Data Generation

To compare estimates based on the sum–score approach and the IRT approach, 150 datasets consisting of item-level twin data obtained at two time points were generated under different scenarios. While additive genetic and unique-environmental variances were fixed to 0.80 and 0.20, the magnitude of covariance explained by these two sources was manipulated, leading to covariances matrices equal to $\Sigma_{A}=\left[\begin{array}{cc} 0.80 & X\\ X & 0.80 \end{array}\right]$ and $\Sigma_{E}=\left[\begin{array}{cc} 0.20 & X\\ X & 0.20 \end{array}\right]$ where *X* was set to either 0.16, 0.40 or 0.60 (in Σ***_A_***) and 0.04, 0.10 or 0.15 (in Σ**_E_**) to obtain a correlation equal to respectively 0.20, 0.50, and 0.80. We considered all possible combinations, resulting in a total of nine different scenarios. In this simulation study, the data was simulated under the AE model meaning that the variance was only decomposed into components due to additive genetic influences and unique-environmental influences. In every scenario, phenotypic means at both time points, μ_1_ and μ_2_, were simulated to be equal to zero. The Rasch model was used to simulate responses to dichotomous items at both time points. Item parameters were simulated once from a normal distribution with a mean of zero and a standard deviation of one and then used for the data generation in every condition at both time points (assuming measurement invariance). In every scenario, the number of items was fixed to 20 and the total number of twin pairs to 1,500, consisting of 1,080 (72% of total *N*) DZ twin pairs and 480 (28% of total *N*) MZ twin pairs. This particular ratio was chosen to approximately reflect the ratio of MZ and DZ twins typically found in European twin registers. This resulted in reliability estimates similar to the ones we found for the empirical data (for more details see the data application section). Cronbach’s alpha was equal to 0.77 and 0.78 at the first and second time point respectively and (Spearman Brown corrected) split-half reliability was equal to 0.79 at both time points.

### Data Analyses

The simulated data were then analyzed on the basis of the sum–scores approach and the IRT approach. For the IRT-based analysis, item-level data was analyzed using the Rasch model where the phenotypic mean of the first time point was fixed to zero (e.g., μ_1_ = 0) to identify the model.

Under the sum–score approach, the item scores (e.g., answered at the first and second time point respectively) were added up to create two sum–scores (e.g., for the first and second time point). Then, instead of performing the AE decomposition based on the θ value of every twin *j* (see Equations 6 and 7 for MZ twins and Equations 12 and 13 for DZ twins) as is done in the latent state model, the decomposition was done on the sum–scores directly. For MZ twins, this yields:

(19) $$\text{Twin }1:\;\begin{array}{c} S_{i11}\\ S_{i12} \end{array}∼MVN\left(\left(\begin{array}{c} \mu_{1}+A1_{i}\\ \mu_{2}+A2_{i} \end{array}\right)\;,\;\Sigma_{E}\right)\;$$

(20) $$\text{Twin }2:\;\begin{array}{c} S_{i21}\\ S_{i22} \end{array}∼MVN\left(\left(\begin{array}{c} \mu_{1}+A1_{i}\\ \mu_{2}+A2_{i} \end{array}\right)\;,\;\Sigma_{E}\right)\;$$

where *S* *_i_*_11_ and *S* *_i_*_12_ denote the phenotypic trait of the first twin of family *i* at time point *t* (here: two time points), respectively and are calculated by adding up the item scores of that twin at the particular time point (e.g., for the first time point and the first twin from family *i*, $S_{i11}=\sum_{1}^{K}Y_{i11k}$ where *Y* *_i_*_11_ *_k_* denotes the answer of the first twin of family *i* to item *k* at time point 1 and *K* denotes the total number of items). The same logic applies to DZ twins, with the only exception that the expected value of the multivariate distribution includes the unique additive genetic effect of every individual twin *j* from family *i* (see Equations 12 and 13). The sum–scores were then analyzed using the same JAGS script but instead of estimating a Rasch model, the decomposition into additive genetic and unique-environmental influences took place directly on the sum–scores (ignoring measurement error) as described above. Furthermore, instead of fixing the phenotypic mean at the first time point to zero, a normal prior distribution was used (e.g., μ_1_ **∼** *N*(0, 10)). The JAGS script that was used can be found in the online **Supplementary Material**.

As advised in the statistical literature on longitudinal models (see e.g. Moeller, 2015), sum–scores were not standardized but we analyzed the raw sum–scores as they were simulated. Consequently, sum–score analysis and IRT analysis were performed on different measurement scales and results were not comparable when not standardized (e.g., estimates of variances and covariances could not be directly compared). Therefore, we calculated standardized measures for all replicated data sets, consisting of heritability estimates at both times ($h_{T1}^{2}$ and $h_{T2}^{2}$) and the correlation between additive genetic [ρ(*A*1, *A*2)] and unique-environmental [ρ(*E*1, *E*2)] influences. Comparable to the ACE model, heritability was defined as the proportion of the total phenotypic variance that can be explained by additive genetic variance (see Equations 14 and 15). Note that the total variance at both time points, $\sigma_{P1}^{2}$ and $\sigma_{P2}^{2}$ respectively, is composed of only two sources in the AE decomposition model (e.g., $\sigma 2_{P1}=\sigma_{A1}^{2}+\sigma 2_{E1}$ and $\sigma_{P2}^{2}=\sigma_{A2}^{2}+\sigma_{E2}^{2}$), resulting in a simulated heritability equal to 0.80 at both time points ($h_{T1}^{2}=h_{T2}^{2}=0.80$). Averaged over 150 replications, the posterior means and standard deviations were calculated for all standardized measures. We also calculated the standard deviation of all posterior means of standardized measures, which can be compared to standard errors in terms of frequentist statistics.

The open-source software package R (R Development Core Team, 2008) was used to simulate data and analyze the results of the simulation study. The MCMC estimation was done in the freely obtainable MCMC program JAGS (Plummer, 2003) and as an interface from R to JAGS, we used the rjags package (Plummer, 2003). The JAGS script that was used can be found in the online **Supplementary Material**. With minor adoptions, this script can also be used in the free software packages WinBUGS (Lunn et al., 2000) or OpenBugs (Thomas et al., 2006). After a burn-in phase of 20,000 iterations, the characterization of the posterior distribution for the model parameters was based on an additional 15,000 iterations from one Markov chain. The burn-in period was chosen based on earlier test runs with multiple chains and calculating the Gelman and Rubin diagnostic (Gelman and Rubin, 1992).

### Results

Posterior point estimates and standard deviations of all relevant parameters, averaged over 150 replications, can be found in **Tables 1**–**3**. For every parameter, the standard deviation of posterior means (e.g., comparable to a standard error in frequentist statistics) can be found in the third row.

**Table 1:** Results of simulation study 1: *ρ* (A1, A2) fixed to 0.2 while *ρ* (E1, E2) is equal to 0.2, 0.5 and 0.8.

	ρ (*A*1, *A*2) = 0.2; ρ (*E*1, *E*2)* = 0.2				ρ (*A*1, *A*2) = 0.2; ρ (*E*1, *E*2)* = 0.5				ρ (A1, A2) = 0.2; ρ (*E*1, *E*2)* = 0.8			
	$h_{T1}^{2}$	$h_{T2}^{2}$	ρ (*A*1, *A*2)	ρ (*E*1, *E*2)	$h_{T1}^{2}$	$h_{T2}^{2}$	ρ (*A*1, *A*2)	ρ (*E*1, *E*2)	$h_{T1}^{2}$	$h_{T2}^{2}$	ρ (*A*1, *A*2)	ρ (*E*1, *E*2)
True value	0.80	0.80	0.20	0.20	0.80	0.80	0.20	0.50	0.80	0.80	0.20	0.80
Sum–scores	0.62 (0.02)	0.62 (0.02)	0.19 (0.04)	0.09 (0.04)	0.62 (0.02)	0.62 (0.02)	0.20 (0.04)	0.21 (0.04)	0.62 (0.02)	0.62 (0.02)	0.20 (0.04)	0.30 (0.04)
	0.03	0.02	0.04	0.04	0.03	0.03	0.04	0.04	0.03	0.03	0.04	0.04
IRT	0.79 (0.03)	0.79 (0.03)	0.20 (0.04)	0.21 (0.10)	0.79 (0.03)	0.79 (0.03)	0.21 (0.04)	0.45 (0.09)	0.79 (0.03)	0.79 (0.03)	0.22 (0.04)	0.62 (0.07)
	0.03	0.03	0.04	0.09	0.03	0.03	0.04	0.08	0.03	0.03	0.04	0.06

Average posterior means (SD) averaged over 150 replications. Third line: Standard deviation of posterior means. Asterisk signifies the parameter value that was changed between simulation conditions.

**Table 2:** Results of simulation study 1: *ρ* (A1, A2) fixed to 0.5 while *ρ* (E1, E2) is equal to 0.2, 0.5 and 0.8.

	ρ (*A*1, *A*2) = 0.5; ρ (*E*1, *E*2)* = 0.2				ρ (*A*1, *A*2) = 0.5; ρ (*E*1, *E*2)* = 0.5				ρ (*A*1, *A*2) = 0.5; ρ (*E*1, *E*2)* = 0.8			
	$h_{T1}^{2}$	$h_{T2}^{2}$	ρ (*A*1, *A*2)	ρ (*E*1, *E*2)	$h_{T1}^{2}$	$h_{T2}^{2}$	ρ (*A*1, *A*2)	ρ (*E*1, *E*2)	$h_{T1}^{2}$	$h_{T2}^{2}$	ρ (*A*1, *A*2)	ρ (*E*1, *E*2)
True value	0.80	0.80	0.50	0.20	0.80	0.80	0.50	0.50	0.80	0.80	0.50	0.80
Sum–scores	0.62 (0.02)	0.62 (0.02)	0.50 (0.03)	0.07 (0.04)	0.62 (0.02)	0.62 (0.02)	0.50 (0.03)	0.21 (0.04)	0.62 (0.02)	0.62 (0.02)	0.50 (0.03)	0.31 (0.04)
	0.03	0.03	0.03	0.04	0.02	0.03	0.03	0.04	0.02	0.03	0.03	0.04
IRT	0.79 (0.03)	0.79 (0.03)	0.51 (0.03)	0.17 (0.10)	0.79 (0.03)	0.79 (0.03)	0.51 (0.03)	0.45 (0.08)	0.78 (0.03)	0.78 (0.03)	0.52 (0.03)	0.64 (0.07)
	0.03	0.03	0.03	0.09	0.03	0.03	0.03	0.08	0.03	0.03	0.03	0.05

Average posterior means (SD) averaged over 150 replications. Third line: Standard deviation of posterior means. Asterisk signifies the parameter value that was changed between simulation conditions.

**Table 3:** Results of simulation study 1: *ρ* (A1, A2) fixed to 0.8 while *ρ* (E1, E2) is equal to 0.2, 0.5 and 0.8.

	ρ (*A*1, *A*2) = 0.8; ρ (*E*1, *E*2)* = 0.2				ρ (*A*1, *A*2) = 0.8; ρ (*E1*, *E2*)* = 0.5				ρ (*A*1, *A*2) = 0.8; ρ (*E*1, *E*2)* = 0.8			
	$h_{T1}^{2}$	$h_{T2}^{2}$	ρ (*A*1, *A*2)	ρ (*E*1, *E*2)	$h_{T1}^{2}$	$h_{T2}^{2}$	ρ (*A*1, *A*2)	ρ (*E*1, *E*2)	$h_{T1}^{2}$	$h_{T2}^{2}$	ρ (*A*1, *A*2)	ρ (*E*1, *E*2)
True value	0.80	0.80	0.80	0.20	0.80	0.80	0.80	0.50	0.80	0.80	0.80	0.80
Sum–scores	0.62 (0.02)	0.62 (0.02)	0.76 (0.03)	0.07 (0.04)	0.62 (0.02)	0.62 (0.02)	0.76 (0.03)	0.20 (0.04)	0.62 (0.02)	0.62 (0.02)	0.76 (0.02)	0.31 (0.04)
	0.02	0.02	0.03	0.04	0.02	0.02	0.02	0.04	0.02	0.03	0.03	0.04
IRT	0.80 (0.03)	0.79 (0.03)	0.76 (0.03)	0.18 (0.09)	0.80 (0.03)	0.79 (0.03)	0.76 (0.02)	0.46 (0.08)	0.78 (0.03)	0.79 (0.03)	0.77 (0.02)	0.65 (0.06)
	0.03	0.03	0.03	0.09	0.03	0.03	0.02	0.08	0.03	0.03	0.02	0.06

Average posterior means (SD) averaged over 150 replications. Third line: Standard deviation of posterior means. Asterisk signifies the parameter value that was changed between simulation conditions.

As the difference in phenotypic means at both time points (e.g. μ*_T_*_1_ – μ*_T_*_2_) as well as difficulty parameters (e.g., β*_k_*) were very close to their true values in every scenario, estimates are not displayed here but can be obtained from the first author.

Heritability estimates were underestimated at both time points when the sum–score approach was used. This bias was very consistent: Regardless of scenario or time point, the heritability estimate was equal to 0.62 opposed to the true value of 0.80. When the IRT approach was used, heritability estimates were either equal to the true value or the difference between simulated value and estimated value was negligibly small. The largest bias was equal to 0.02 in the conditions in which both ρ (*A*1, *A*2) and ρ (*E*1, *E*2) were large.

When ρ (*A*1, *A*2) was simulated to be equal to 0.2, there was a small bias in the estimates of the sum–score approach when ρ (*E*1, *E*2) was equal to 0.2 and a small bias in the results of the IRT approach when ρ (*E*1, *E*2) was equal to 0.5 or 0.8. In both cases, however, this bias was negligibly small [e.g., ρ (*A*1, *A*2) was estimated 0.19 instead of 0.20 in case of the sum–score approach and 0.21 and 0.22 respectively when the IRT approach was used]. When the true value of ρ (*A*1, *A*2) was equal to 0.5, there was no bias in the sum–score approach and a small bias in the IRT approach [e.g., 0.51 and 0.52 instead of the simulated value of ρ (*E*1, *E*2) = 0.50]. When ρ (*A*1, *A*2) was simulated to be equal to 0.80, the amount of bias in both approaches was more severe and fairly comparable (e.g., in most cases, the estimated value was approximately 0.76).

There was bias in the estimates of ρ (*E*1, *E*2) in both approaches, but this bias was clearly more severe when the sum–score approach was used. For example, when the true value of ρ (*A*1, *A*2) was equal to 0.2, the estimated values were respectively 0.08 when the sum–score approach was used and 0.21 when the IRT approach was used. For both approaches, this bias increased with increasing correlation among unique-environmental influences: When the true value was equal to 0.5, the estimates were equal to 0.21 (sum–score approach) and 0.45 (IRT approach) respectively and equal to 0.30 (sum–score approach) and 0.62 (IRT approach) when the correlation was simulated to be equal to 0.8. Here and there, this bias was larger or smaller, but overall, the same pattern could be observed when ρ (*A*1, *A*2) was simulated to be equal to either 0.5 or 0.8.

## Simulation Study 2

Based on the results of the first simulation study, in the second simulation study, the worst case scenario (e.g., the combination of correlations among additive genetic and unique-environmental influences that lead to the largest bias) was selected and, under this scenario, an ACE model was used for the data generation. In order to investigate the performance of the ACE model, we manipulated the magnitude of the covariance that can be explained by common-environmental influences, $\sigma_{CC}^{2}$

### Data Generation

To have reasonable values, in this simulation study, variance explained by additive genetic influences was set to 0.6 at both time points (e.g., $\sigma_{A1}^{2}=\sigma_{A2}^{2}=0.6$) and variance due to common-environmental and unique-environmental influences to 0.2 at both time points (e.g., $\sigma_{C1}^{2}=\sigma_{C2}^{2}=\sigma_{E1}^{2}=\sigma_{E2}^{2}=0.2$), leading to variance–covariance matrices equal to $\Sigma_{A}=\left[\begin{array}{cc} 0.60 & 0.12\\ 0.12 & 0.60 \end{array}\right]$, $\Sigma_{C}=\left[\begin{array}{cc} 0.20 & X\\ X & 0.20 \end{array}\right]$ and $\Sigma_{E}=\left[\begin{array}{cc} 0.20 & 0.16\\ 0.16 & 0.20 \end{array}\right]$ where *X* was set to either 0.04, 0.10, or 0.16 to create a correlation of 0.2, 0.5, and 0.8 between common-environmental influences at the different time points. As in the first simulation study, under every scenario, 150 datasets were simulated. The number of items was fixed to 20 and the Rasch model was used to simulate responses to dichotomous items at both time points. The same difficulty parameters were used as in the first simulation study for every scenario and every time point. Due to the higher complexity of the ACE model, the total number of twins was increased to a total of 3,000 but the ratio between MZ and DZ twin pairs was the same as in the first simulation study (e.g., 2160 (72% of total *N*) DZ twin pairs and 840 (28% of total *N*) MZ twin pairs). Again, the phenotypic mean was assumed to be equal to zero at both time points and the same item parameters were used to simulate the item data. Cronbach’s alpha was equal to 0.79 and 0.78 at the first and second time point respectively and (Spearman Brown corrected) split-half reliability was equal to 0.80 at both time points.

### Data Analysis

As in the first simulation study, the simulated data was analyzed with both the IRT approach and the sum–score approach. For the IRT-based analysis, item-level data was analyzed using the Rasch model where the first time point was fixed to 0 to identify the model. Under the sum–score approach, sum–scores were calculated for the first and second time point respectively. Instead of performing the ACE decomposition based on the θ values of every twin *j* (see Equations 6 and 7 for MZ twins and Equations 12 and 13 for DZ twins) as is done in the latent state model, the decomposition was done on the sum–scores directly. Comparable to Equations 19 and 20, for MZ twins, this yields:

(21) $$\begin{array}{c} S_{i11}\\ S_{i12} \end{array}∼MVN\left(\left(\begin{array}{c} \mu_{1}+C1_{i}+A1_{i}\\ \mu_{2}+C2_{i}+A2_{i} \end{array}\right)\;,\;\Sigma_{E}\right)\;\text{ Twin }1$$

(22) $$\begin{array}{c} S_{i21}\\ S_{i22} \end{array}∼MVN\left(\left(\begin{array}{c} \mu_{1}+C1_{i}+A1_{i}\\ \mu_{2}+C2_{i}+A2_{i} \end{array}\right)\;,\;\Sigma_{E}\right)\;\text{ Twin }2$$

where *S* *_i_*_11_ and *S* *_i_*_12_ represent the phenotypic trait of the first twin of family *i* at time point *t* (here: two time points) respectively and are calculated by adding up the item scores of that twin at the particular time point (e.g., for the first time point and the first twin from family *i*, $S_{i11}=\sum_{1}^{K}Y_{i11k}$ where *Y* *_i_*_11_ *_k_* denotes the answer of the first twin of family *i* to item *k* at the first time point and *K* denotes the total number of items). The same logic applies to DZ twins, but here the expected value of the multivariate normal distribution included the unique additive genetic effects of every individual twin *j* from family *i* (see Equations 12 and 13). The sum–scores were then analyzed using the same JAGS script but instead of estimating a Rasch model, sum–scores were directly decomposed into additive genetic, common-environmental, and unique-environmental influences. Instead of fixing the phenotypic mean at the first time point to zero (e.g., μ_1_ = 0), a normal prior distribution was used [e.g., μ_1_ **∼** *N*(0, 10)]. Both JAGS scripts can be found in the online **Supplementary Material**.

For every scenario, averaged over 150 replications, the posterior means and standard deviations were calculated for all standardized measures [e.g., $h_{T1}^{2}$, $h_{T2}^{2}$, ρ (*A*1, *A*2), ρ (*C*1, *C*2) and ρ (*E*1, *E*2)]. We also calculated the standard deviation of all posterior means of standardized measures, which can be compared to standard errors in terms of frequentist statistics. Heritability was defined as the proportion of the total phenotypic variance that can be explained by additive genetic variance (see Equations 14 and 15). As in the first simulations study, we used the open-source software package R (R Development Core Team, 2008) to simulate the data and analyze the results, the open-source MCMC program JAGS (Plummer, 2003) for the MCMC estimation and the rjags package (Plummer, 2013) as an interface from R to JAGS. The JAGS script that was used can be found in the online **Supplementary Material**, which can also be used in the free software packages WinBUGS (Lunn et al., 2000) or OpenBugs after minor adoptions. As in the first simulation study, a burn-in period of 20,000 iterations was used and the characterization of the posterior distribution for the model parameters was based on an additional 15,000 iterations from 1 Markov chain. This was chosen based on earlier test runs with multiple chains and calculating the Gelman and Rubin diagnostic (Gelman and Rubin, 1992).

### Results

Posterior point estimates and standard deviations of all relevant parameters, averaged over 150 replications, can be found in **Table 4**. For every parameter, the standard deviation of posterior means (e.g., comparable to a standard error in frequentist statistics) can be found in the third row.

**Table 4:** Results of simulation study 2.

	ρ (*C*1, *C*2) = 0.2					ρ (*C*1, *C*2) = 0.5					ρ (*C*1, *C*2) = 0.8				
	$h_{T1}^{2}$	$h_{T2}^{2}$	ρ (*A*1, *A*2)	ρ (*C*1, *C*2)	ρ (*E*1, *E*2)	$h_{T1}^{2}$	$h_{T2}^{2}$	ρ (*A*1, *A*2)	ρ (*C*1, *C*2)	ρ (*E*1, *E*2)	$h_{T1}^{2}$	$h_{T2}^{2}$	ρ (*A*1, *A*2)	ρ (*C*1, *C*2)	ρ (*E*1, *E*2)
True value	0.60	0.60	0.20	0.20	0.80	0.60	0.60	0.20	0.50	0.80	0.60	0.60	0.20	0.80	0.80
Sum–scores	0.56 (0.03)	0.50 (0.05)	0.21 (0.05)	0.21 (0.16)	0.32 (0.03)	0.55 (0.03)	0.48 (0.05)	0.23 (0.06)	0.23 (0.13)	0.31 (0.03)	0.51 (0.04)	0.47 (0.05)	0.24 (0.06)	0.24 (0.09)	0.32 (0.03)
	0.03	0.05	0.06	0.06	0.03	0.03	0.06	0.06	0.06	0.03	0.04	0.05	0.06	0.06	0.03
IRT	0.65 (0.04)	0.56 (0.06)	0.24 (0.06)	0.25 (0.06)	0.65 (0.05)	0.63 (0.05)	0.56 (0.06)	0.28 (0.06)	0.55 (0.06)	0.65 (0.06)	0.58 (0.05)	0.55 (0.06)	0.30 (0.06)	0.61 (0.06)	0.65 (0.05)
	0.03	0.06	0.04	0.08	0.04	0.04	0.06	0.05	0.09	0.04	0.05	0.05	0.06	0.08	0.05

*ρ*(A*1*, A2) fixed to 0.2 and *ρ*(E1, E2) to 0.8 while *ρ*(C1, C2) is equal to 0.2, 0.5 and 0.8. Average posterior means (SD) averaged over 150 replications. Third line: Standard deviation of posterior means.

Since the difference between the phenotypic means (e.g., μ*_T_*_1_ – μ*_T_*_2_) and the item difficulty parameters (e.g., β*_k_*) were very close to their true values in all scenarios, results are not displayed here but can be obtained from the first author.

Both approaches resulted in biased heritability estimates at both time points. As could be expected based on the results from the first simulation study, in most conditions, heritability was underestimated when the sum–score approach was used and the bias was larger than under the IRT approach. A surprising exception to this is the condition where ρ (*C*1, *C*2) was simulated to be equal to 0.20, where the bias in $h_{T1}^{2}$ was smaller for the sum–score approach than for the IRT approach. Note however that the difference is fairly minor (e.g., $h_{T1}^{2}$ was simulated to be equal to 0.60 and estimated 0.56 under the sum–score approach and 0.65 when the IRT approach was used).

Concerning estimates of ρ (*A*1, *A*2), surprisingly, the sum–score approach resulted in less bias than the IRT approach. The bias was the largest when ρ (*C*1, *C*2) was simulated to be equal to 0.20 (e.g., 0.30 under the IRT approach and 0.24 under the sum–score approach). Estimates of ρ (*C*1, *C*2) were generally less biased when the IRT approach was used, except when ρ (*C*1, *C*2) was simulated to be equal to 0.20. Under both frameworks, the bias was the largest when ρ (*C*1, *C*2) was equal to 0.80 (estimate of 0.24 and 0.61 for the sum–score and IRT approach respectively). Estimates of ρ (*E*1, *E*2), were biased under both approaches with less bias when the IRT approach was used. The severity of this bias was independent of the magnitude of the correlation.

## Application

To further illustrate the differences and similarities between the classical approach (e.g., using sum–scores) and the psychometric approach (e.g., analyzing item-level data), we applied both approaches to longitudinal twin data consisting of two measurement occasions.

### Data

As part of a much larger study (see e.g. Posner et al., 1996) conducted in Australia in 1980 (first wave) and 1988 to 1990 (second wave), the dataset contained twins’ item answers on a scale developed by Wilson and Patterson (1968) that measures social attitudes related to the conservatism by means of 28 items. The total sample size was 8,016 twin pairs of which 4,541 were DZ twin pairs and 3,475 MZ twin pairs. Mean age was 31.61 (*SD* = 14.28, range of 13–96) at the second wave and twins were 8 years younger at the first wave (e.g., mean age of 23.61). The scale consists of very short catch-phrases, as for example “Liberals” and “Living together.” The test taker is given a list of these catch-phrases and is instructed as follows: “Please indicate whether or not you agree with each topic by circling “Yes” or “No” as appropriate. If uncertain, please circle “?.”

For a direct application of the model presented here, answer categories were recoded as 0 (“No”), missing (“?”) and 1 (“Yes”) to get dichotomously scored item data suitable for analysis under the Rasch model. Note however that IRT models that can handle ordinal data such as the partial credit model (PCM) or the graded response model (GRM), might be more suitable for the data at hand (see Schwabe et al., 2016). Before analyzing the data, a factor analysis and reliability analysis was performed to select items that form together a unidimensional and reliable scale. For the factor analysis, the psych rpackage (Revelle, 2018) was used which allows performing an exploratory factor analysis on dichotomous data based on the polychoric correlations among the individual items. Based on the results, a total of 16 items was selected. Four of these items were recoded since they showed a negative correlation with the sum–score. This resulted in a unidimensional scale with Cronbach’s alpha equal to 0.75 and (Spearman Brown corrected) split-half reliability equal to 0.81 and 0.82 at the first and second time point respectively.

### Analysis

To determine what genetic model fitted the data well, while, at the same time, being parsimonious, we first run both an ACE and AE model under the IRT approach and calculated the deviance information criterion (DIC, Spiegelhalter et al., 2002). The DIC is a measure that estimates the amount of information that is lost when a given model is used to present the process that generates the data while taking into account both goodness of fit and complexity of the particular model. Based on the results, the AE model was chosen and also analyzed under the sum–score approach. Under both approaches, we assumed that the missing data was missing at random (MAR). Under the IRT approach, we assumed measurement invariance.

For the data cleaning and analysis of results, we used the statistical software package R (R Development Core Team, 2008). The MCMC estimation was done in JAGS (Plummer, 2003) and the rjags package (Plummer, 2013) was used as a pipeline between JAGS and R. For both approaches, we used the same prior distributions and scripts as in the simulation study (see the online **Supplementary Material**). As in the simulation study, we chose the number of burn-in iterations that were needed to draw from the posterior distribution on earlier test runs with multiple chains and calculating the Gelman and Rubin diagnostic.

#### Sum–Score Approach

**Figure 1:**
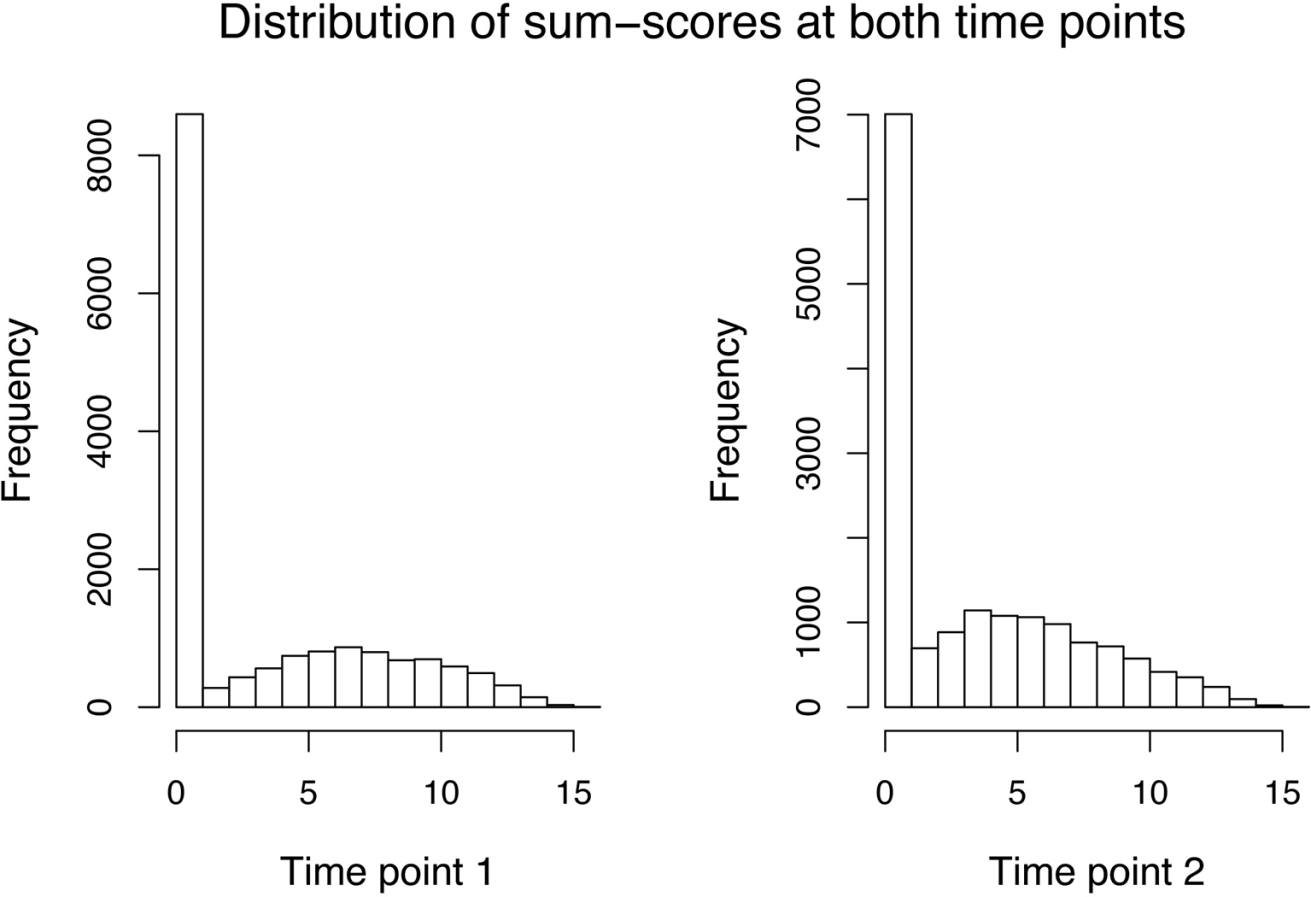
Data application: Distribution of the sum–scores of both MZ and DZ twins at the first (left side) and second (right side) time point.

For the sum–score approach, we had to calculate the sum–score for every twin by adding up his or her answer on every item. As simple imputation methods (e.g., imputing all missing responses of an item by its mean or mode of the available responses) resulted in quite peaked distributions and will likely result in bias, we decided to calculate an individual’s twin sum–score based on all non-missing answers. The variance decomposition was then done directly on the sum–score (see Equations 19 and 20). The distribution of sum–scores (MZ and DZ twins) at both time points can be seen in **Figure 1**. After an adoption phase of 5,000 iterations and 22,000 burn-in period iterations, the characterization of the posterior distribution was based on 15,000 additional iterations from 1 chain.

#### IRT Approach

Using the IRT approach, we were able to analyze the data on item-level such that we could immediately use the raw data as input in JAGS (Plummer, 2003) without having to impute data first. JAGS automatically imputes the missing item data from the parameter estimates (e.g., as derived from the observed outcomes) at every iteration. Note also that the IRT approach uses a different missing data treatment than the sum–score approach. This will impact the results additional to the different ways of dealing with measurement error. After an adoption phase of each 5,000 iterations and 5,000 burn-in period iterations, the characterization of the posterior distribution was based on 15,000 additional iterations from 1 chain.

Under both approaches, we calculated heritability at both time points, $h_{T1}^{2}$ and $h_{T2}^{2}$ the correlation between additive genetic influences between additive genetic influences [e.g., ρ (*A*1, *A*2)] as well as the correlation between unique-environmental influences [e.g., ρ (*E*1, *E*2)] and the phenotypic stability, defined as the correlation between observed sum–scores at both time points (sum–score approach) and as the correlation between latent trait scores at both time points (IRT) approach. For every parameter, we also calculated the 95% highest posterior density interval (HPD, see, e.g., Bolstad, 2007), using the rpackage BayesTwin (Schwabe, 2017). The HPD can be seen as the Bayesian counterpart of a frequentist confidence interval.

Heritability was defined as the proportion of the phenotypic variance (at the respective time point) that can be explained by additive genetic variance and was calculated as $h_{T1}^{2}=\frac{\sigma_{A1}^{2}}{\sigma_{P1}^{2}}=\frac{\sigma_{A1}^{2}}{\sigma_{A1}^{2}+\sigma_{E1}^{2}}$ for the first time point and as $h_{T2}^{2}=\frac{\sigma_{A2}^{2}}{\sigma_{P2}^{2}}=\frac{\sigma_{A2}^{2}}{\sigma_{A2}^{2}+\sigma_{E2}^{2}}$ for the second time point.

### Application Results

Estimated heritability at both time points (e.g., $h_{T1}^{2}$ and $h_{T2}^{2}$), estimated correlation between additive genetic influences [e.g., ρ(*A*1, *A*2)] and between unique-environmental influences [e.g., ρ(*E*1, *E*2)] are displayed in **Table 5** for both the IRT-based (first part of the table) and sum–score based (second part of the table) analysis. In the first row of every analysis part, the posterior mean and standard deviation is displayed and in the second row the 95% highest posterior density interval (HPD, see, e.g., Bolstad, 2007). The IRT approach resulted in a much higher estimate of phenotypic stability than the sum–score approach: The correlation between observed sum–scores at both time points (sum–score approach) was equal to 0.50 and the correlation between latent trait scores at both time points (IRT) was equal to 0.95.

**Table 5:** Longitudinal analysis of social attitudes related to conservatism. Results of the latent state model and the sum–score approach.

	$h_{T1}^{2}$	$h_{T2}^{2}$	ρ (*A*1, *A*2)	ρ (*E*1, *E*2)
IRT-based analysis:				
Posterior mean (SD)	0.85 (0.01)	0.84 (0.01)	0.95 (0.01)	0.83 (0.03)
95% HPD	[0.83;0.88]	[0.81;0.86]	[0.94;0.97]	[0.77;0.88]
Sum–score based analysis:				
Posterior mean (SD)	0.85 (0.01)	0.68 (0.01)	0.58 (0.01)	0.23 (0.01)
95% HPD	[0.85;0.88]	[0.66;0.69]	[0.56;0.59]	[0.20;0.26]

Posterior mean (standard deviation) and the 95% HPD of the variance components and of the inter-temporal correlations for the AE model at two time points. Based on N = 8,016 twin pairs (4,541 DZ twin pairs and 3,475 MZ twin pairs).

It can be seen that heritability estimates were the same for the first time point under both approaches. The IRT approach however resulted in a higher heritability estimate at the second time point (e.g., 68% and 85% respectively). By using a longitudinal design instead of two cross-sectional analyses, we can also draw conclusions on the (relative importance of) additive genetic and unique-environmental influences in creating covariance between the personality measures at the two waves. Both approaches resulted in higher estimates of ρ (*A*1, *A*2) compared to ρ (*E*1, *E*2), meaning that additive genetic influences were more important in creating covariance between the two time points. However, estimates were generally higher under the IRT approach [e.g., ρ (*A*1, *A*2) = 0.95 and ρ (*E*1, *E*2) = 0.83] than under the sum–score approach [e.g., ρ (*A*1, *A*2) = 0.58 and ρ (*E*1, *E*2) = 0.23].

Note that the IRT approach leads to different conclusions than the sum–score approach. While the IRT-based analysis resulted in comparable estimates at both time points, the sum–score approach resulted in a lower heritability estimate at the second wave, suggesting that additive genetic influences were more important in creating individual differences at an earlier age than at a later age. Furthermore, the results of the IRT approach suggested that both, additive genetic and unique-environmental influences, were important in explaining covariance among the two waves (e.g., both estimates are above 0.80). Contrarily, the sum–score based results indicated that additive genetic influences were more important in explaining covariance between the two waves than unique-environmental influences [e.g., with ρ (*A*1, *A*2) being almost twice as big as ρ (*E*1, *E*2)].

## Discussion

Earlier research has shown that heritability estimates of univariate twin analyses can be biased when questionnaire items are aggregated into a single score such as is done in the commonly used sum–score approach. van den Berg et al. (2007) illustrated this potential bias and showed that the problem can be solved by, instead, analyzing item-level data through integrating an explicit measurement item response theory (IRT) model into the twin model. However, these results only apply to the case where the phenotype was measured at a single occasion. Here, we investigated how these results generalize to the longitudinal twin design, where the phenotype is assessed at multiple time points. Besides heritability estimates at all time points, this design allows us to estimate the magnitude of the covariance across time that can be explained by additive genetic and environmental influences respectively. After introducing a latent state longitudinal twin A(C)E model that integrates a Rasch IRT model into the longitudinal twin design, in this paper, we investigated 1) how large the bias is when the sum–score approach is used in the longitudinal design, and 2) if we can solve the potential bias by using the latent state longitudinal model. To this end, two simulation studies were conducted.

In the first simulation study, a fixed number of twin pairs and items was simulated while the magnitude of covariance that was due to additive genetic- and unique-environmental influences was manipulated. Since the the latent state longitudinal twin model is computationally demanding, we used a simple AE model in the first simulation study which allowed us to simulate and analyze a broader range of scenarios. As expected, results showed that heritability estimates were underestimated when the sum–score approach was used compared to only little bias when the simulated data was analyzed with the latent state model. Surprisingly, under some conditions, estimates of the sum–score approach concerning the correlation between additive genetic influences at both time points [e.g., ρ (*A*1, *A*2)] were closer to the simulated values than the estimates of the IRT-based approach. This bias was, however, only small. Regarding covariance that can be explained by unique-environmental influences, there was bias in both approaches. The severity of this bias was clearly larger in the sum score approach, but not negligibly low in the IRT approach either. This bias increased with increasing magnitude of the correlation. A similar picture emerges in the field of multitrait–multimethod research. Here, it has been shown that the correlated trait- correlated-methods model (Jöreskog, 1974; Widaman, 1985), which technically resembles the latent state twin model, is affected by identification and estimation problems, especially when trait and method factors are highly correlated among each other (Marsch, 1989; Marsh and Bailey, 1991; Kenny and Kashy, 1992; Marsh et al., 1992). Translated to the case of the latent state twin model, this means that estimation problems are to be expected when the additive genetic- and unique-environmental influences are rather stable across time.

Based on the results of the first simulation study, the worst case scenario was selected (e.g., the one with the largest bias) and then this scenario was replicated in the second simulation study while the magnitude of covariance between the two points due to common-environmental influences was manipulated [e.g., estimating an ACE model with varying ρ (*C*1, *C*2)]. As in the first simulation study, heritability at both time points was underestimated when the sum–score approach was used. Although there was more bias under the sum–score approach, compared to the first simulation study, there was also non-negligible bias in the heritability estimates when the IRT approach was used. While ρ (*A*1, *A*2) and ρ (*E*1, *E*2) were less biased when the IRT approach was used, surprisingly, there was less bias in the results of the sum–score approach in estimates of ρ (*C*1, *C*2). This bias can be related to our choice of prior distributions. Although the inverse Wishart distribution is a common choice for variance-covariance matrices (van Erp et al., 2018), research has shown that this prior setting also comes with disadvantages, such that the uncertainty for all parameters of the variance–covariance matrix is controlled by a single degree of freedom parameter (Gelman et al., 2004). One of the proposed alternatives to overcome these problems consists of using hierarchical inverse Wishart priors where hyperpriors are used, meaning that also for the diagonal elements of the scale matrix (Huang and Wand, 2013) or both the diagonal elements of the scale matrix and the degrees of freedom of the inverse Wishart prior (Bourina and Feron, 2013), prior distributions are used. In an additional simulation study, we investigated the effect of a couple of different prior settings where we directly followed the approach by Ulitzsch et al. (2019) who provide JAGS syntax for a different but similar application. However, the model would not converge (even after 500,000 burn-in iterations), which might have to do with the increase in parameters that had to be estimated. More research is needed to fully explore this option. Details and results of this additional simulation study can be obtained from the first author of this paper.

Even with the presence of some bias, given the available options, we would still argue for the use of latent variable modelling, as it comes with many further advantages not illustrated here (see also Eaves et al., 2005). These include the possibility to treat missing values using sophisticated methods. Also, when some items in a questionnaire change across birth cohorts or across different age range, a sum–score approach may no longer be appropriate, but in many cases, the analysis can still be meaningfully carried out under the IRT framework (van den Berg et al., 2014). Furthermore, earlier research has shown that the use of an aggregated score as the sum–score can lead to the spurious finding of a genotype by environment interaction in case of heterogeneous measurement error while an IRT-based approach is unbiased (Wray et al., 2008; Molenaar and Dolan, 2014; Schwabe and vanden Berg, 2014; Schwabe et al., 2017). A further advantage of the IR approach is the possibility to investigate group differences (e.g., differential item functioning, DIF).

It has to be noted that the simulated data does not necessarily reflect the complex structure seen in real data. For example, in practice, questionnaire data is seldom normally distributed (see e.g. Hertzog and van Alstine, 1990) and questionnaires often consist of only a limited number of items. Gorter et al. (2015) used a simulation study to compare IRT-based plausible value techniques to the results of sum–score based analyses in a multilevel longitudinal design. This design can be seen as a simple form of the longitudinal twin AE decomposition model (e.g., without different genetic correlations and more individuals in every group). In their simulations, they also manipulated the number of questionnaire items, the sample size, and the skewness of the questionnaire data distribution. They found that the difference between both methods becomes consistently larger for the more extreme conditions of the simulation, indicating that IRT-based plausible value techniques are quite robust against more extreme data situations while the bias in the sum–score approach becomes even worse. These results of course do not generalize one by one to a twin design, but based on these results we expect that also in the framework of the longitudinal twin design the IRT approach will be more robust than the sum–score approach.

To illustrate the difference between the classical approach (e.g., using sum–scores) and psychometric genetic modelling (e.g., using an IRT model and analyzing item-level data), we analyzed data of a two-wave twin study, consisting of the answers of 8,016 twins on scale developed to measure social attitudes related to conservatism. The data was analyzed using both approaches, the sum–score approach and the IRT approach. The IRT approach resulted in a higher heritability estimate at the second time point and higher covariance estimates, which is in line with the results of the simulation studies. Also, intuitively, these results make sense: When not taken into account, measurement error will lead to an attenuation of correlations [see also van den Berg et al. (2007) for the univariate case]. While the IRT approach suggested that both, genetic and environmental influences, are important in explaining covariance between the two waves, estimates of the sum–score approach were 1) generally smaller (suggesting less covariance in general) and 2) suggested that additive genetic influences are more important in explaining covariance between the two waves than unique-environmental influences. Note that the two analyses do not only differ in how measurement error was accounted for, but also in how missing values were treated. IRT modeling allows for more sophisticated approaches for dealing with missing data. Of course, in real empirical examples, a more thorough psychometric analysis would be needed. We used a Rasch and AE model here, but the syntax can easily be changed to IRT models that can handle ordinal data (see Schwabe et al., 2016).

A limitation of this paper is that we did not model any residual correlations within twin pairs that cannot be explained by genetic or environmental influences (e.g., accommodated by the application of the ACE model) but are due to model violations of local independence. It is important to note that, depending on the phenotype of interest, this assumption might not hold, since responses of twins of the same twin pair, conditional on the latent variables, can still be correlated due to shared item-specific genetic and environmental influences. Molenaar and Dolan (2014) show how possible violations of conditional independence can be modeled by introducing additional latent variables in the univariate case (e.g., one measurement point). Note however that this will result in a more complex model which potentially introduces estimation problems.

In this paper, we only considered the latent state model for longitudinal genetic data. There are, however, also other longitudinal models such as the genetic simplex model (Boomsma and Molenaar, 1987). The choice of a model depends on the specific hypotheses a researcher has for the data at hand. Regarding the modelling of the phenotype, similar results to those found in this paper are expected for these models. IRT models will surely enable a more sophisticated way to deal with measurement error, missing values, measurement invariance, and changing items.

## Data Availability

All relevant estimates to this study have been made available to the reader in the manuscript or the online supplementary material. The individual data analyzed in this study were obtained from the QIMR Medical Research Institute, collected as part of a much larger study conducted in Australia in 1980 (first wave) and 1988 (second wave). The following restrictions apply: individual twin data are unavailable for public deposition due to data privacy restrictions. Before data can be released, a request must be sent to Prof. dr. Nick Martin from the QIMR Medical Research Institute (nick.martin@qimrberghofer.edu.au).

## Ethics Statement

Methodological article with secondary analyses of data collected by the Australian Twin Register.

## Author Contributions

IS developed the study concept, performed the statistical analyses, and drafted the manuscript. ZG cleaned the data for the empirical application. PH and JT provided critical revisions and feedback on the manuscript. SP supervised the project and provided critical revisions and feedback on both the analysis scripts and the manuscript.

## Conflict of Interest

The authors declare that the research was conducted in the absence of any commercial or financial relationships that could be construed as a potential conflict of interest.
