# Supplementary material for: Psychometric Modelling of Longitudinal Genetically Informative Twin Data
Source: Front Genet. 2019 Oct 16;10:837. doi: 10.3389/fgene.2019.00837 (PMC6807617; doi:10.3389/fgene.2019.00837)
Supplement: Supplementary file 1 [file DataSheet_1.pdf]

Psychometric Modelling of Longitudinal Genetically-Informative Twin Data: Supplementary material

I. Schwabe Z. Gu J. Tijmstra P. Hatemi S. Pohl

This document contains supplementary material for the article "Psychometric Modelling of Longitudinal Genetically-Informative Twin Data".

Below, you can find all JAGS scripts that were used either in the simulation studies or in the data application. All scripts as well as the R script that was used for the data cleaning can also be found online at the personal github page of the first author at `https://github.com/ingaschwabe/Psychometric-Modelling-of-Longitudinal-Twin-Data`.

Longitudinal AE decomposition

IRT approach (Latent trait model)

Following JAGS script fits a longitudinal AE decomposition with an incorporated IRT Rasch model at the phenotypic level (Latent trait AE twin model). The script was used for the first simulation study and the data application.

```
#=============================================================#
# Following JAGS script fits a longitudinal latent trait-state AE twin model
# T = total number of time points (equal to 2 here)
# n_dz = total number of DZ twin families; n_dz = total number of MZ families
# n_items = total number of questionnaire items
#
# Note that this script works for 2 time points but can easily be adjusted
# to work for more time points.
# Any questions: Contact Inga Schwabe (I.Schwabe@uvt.nl)
#=============================================================#

model{
    # Twin model part for MZ twins
    for (fam in 1:n_mz){
        a1_mz[fam,1:T] ~ dmnorm(mu_a[1:T], tau_a[1:T,1:T])

        # Modelling phenotypes:
        pheno_mz_twin1[fam,1:T] ~ dmnorm(mu[1:T] + a1_mz[fam,1:T],
                                         tau_e[1:T,1:T])
        pheno_mz_twin2[fam,1:T] ~ dmnorm(mu[1:T] + a1_mz[fam,1:T],
                                         tau_e[1:T,1:T])

        # IRT part: Rasch model for both time points
        for (k in 1:n_items){
            logit(p_mz_twin1_t1[fam,k]) <- pheno_mz_twin1[fam,1] - beta[k]
            data_mz_twin1_t1[fam,k] ~ dbern(p_mz_twin1_t1[fam,k])

            logit(p_mz_twin2_t1[fam,k]) <- pheno_mz_twin2[fam,1] - beta[k]
            data_mz_twin2_t1[fam,k] ~ dbern(p_mz_twin2_t1[fam,k])
        }

        for (k in 1:n_items){
            logit(p_mz_twin1_t2[fam,k]) <- pheno_mz_twin1[fam,2] - beta[k]
            data_mz_twin1_t2[fam,k] ~ dbern(p_mz_twin1_t2[fam,k])

            logit(p_mz_twin2_t2[fam,k]) <- pheno_mz_twin2[fam,2] - beta[k]
            data_mz_twin2_t2[fam,k] ~ dbern(p_mz_twin2_t2[fam,k])
        }
    }
```

```
40
41          # Twin model part for DZ twins
42      for (fam in 1:n_dz){
43
44          a1_dz[fam,1:T] ~ dmnorm(mu_a[1:T], tau_a[1:T,1:T])
45          a2_dz[fam,1:T] <- a1_dz[fam,1:T]/sqrt(2)
46
47          a3_dz_twin1[fam,1:T] ~ dmnorm(mu_a[1:T], tau_a[1:T,1:T])
48          a3_dz_twin2[fam,1:T] ~ dmnorm(mu_a[1:T], tau_a[1:T,1:T])
49
50          a4_dz_twin1[fam,1:T] <- a3_dz_twin1[fam,1:T]/sqrt(2)
51          a4_dz_twin2[fam,1:T] <- a3_dz_twin2[fam,1:T]/sqrt(2)
52
53          # Modelling phenotypes:
54          pheno_dz_twin1[fam,1:T] ~ dmnorm(mu[1:T] + a2_dz[fam,1:T] +
55                                           a4_dz_twin1[fam,1:T], tau_e[1:T,1:T])
56          pheno_dz_twin2[fam,1:T] ~ dmnorm(mu[1:T] + a2_dz[fam,1:T] +
57                                           a4_dz_twin2[fam,1:T], tau_e[1:T,1:T])
58
59                  # IRT part (Rasch model for both time points):
60          for (k in 1:n_items){
61              logit(p_dz_twin1_t1[fam,k]) <- pheno_dz_twin1[fam,1] - beta[k]
62              data_dz_twin1_t1[fam,k] ~ dbern(p_dz_twin1_t1[fam,k])
63
64              logit(p_dz_twin2_t1[fam,k]) <- pheno_dz_twin2[fam,1] - beta[k]
65              data_dz_twin2_t1[fam,k] ~ dbern(p_dz_twin2_t1[fam,k])
66          }
67
68          for (k in 1:n_items){
69              logit(p_dz_twin1_t2[fam,k]) <- pheno_dz_twin1[fam,2] - beta[k]
70              data_dz_twin1_t2[fam,k] ~ dbern(p_dz_twin1_t2[fam,k])
71
72              logit(p_dz_twin2_t2[fam,k]) <- pheno_dz_twin2[fam,2] - beta[k]
73              data_dz_twin2_t2[fam,k] ~ dbern(p_dz_twin2_t2[fam,k])
74          }
75      }
76
77      # Prior distributions
78      # Normal prior for beta item parameters:
79      for (k in 1:n_items){
80          beta[k] ~ dnorm(0, .1)
81      }
82
83      # The expected value of the first time point is set to 0 to identify the
84      # measurement scale.
85      # A normal prior is set on the remaining time point averages
```

```
86      mu[1] <- 0
87      mu[2] ~ dnorm(0, .1)
88
89      # Inverse Wishart prior distribution for covariance matrices
90      tau_a[1:T,1:T] ~ dwish(omega_a[,], T+1)
91      tau_e[1:T,1:T] ~ dwish(omega_e[,], T+1)
92
93      # Calculate inverse
94      Sigma_a[1:T,1:T] <- inverse(tau_a[,])
95      Sigma_e[1:T,1:T] <- inverse(tau_e[,])
96  }
```

Sum-score approach

Following JAGS script fits a longitudinal AE twin model on sum-scores. The script was used for the first simulation study and the data application.

```
#=============================================================#
# Following JAGS script fits a longitudinal AE twin model using sum-scores
# T = total number of time points
# n_dz = total number of DZ twin families; n_dz = total number of MZ families
#
# Any questions: Contact Inga Schwabe (I.Schwabe@uvt.nl)
#=============================================================#

model{

    # Twin model part for MZ twins
    for (fam in 1:n_mz){
        a1_mz[fam,1:T] ~ dmnorm(mu_a[1:T], tau_a[1:T,1:T])

        # Modelling phenotypes:
        data_mz_twin1[fam,1:T] ~ dmnorm(mu[1:T] + a1_mz[fam,1:T], tau_e[1:T,1:T])
        data_mz_twin2[fam,1:T] ~ dmnorm(mu[1:T] + a1_mz[fam,1:T], tau_e[1:T,1:T])
    }

    # Twin model part for DZ twins
    for (fam in 1:n_dz){

        a1_dz[fam,1:T] ~ dmnorm(mu_a[1:T], tau_a[1:T,1:T])
        a2_dz[fam,1:T] <- a1_dz[fam,1:T]/sqrt(2)

        a3_dz_twin1[fam,1:T] ~ dmnorm(mu_a[1:T], tau_a[1:T,1:T])
        a3_dz_twin2[fam,1:T] ~ dmnorm(mu_a[1:T], tau_a[1:T,1:T])

        a4_dz_twin1[fam,1:T] <- a3_dz_twin1[fam,1:T]/sqrt(2)
        a4_dz_twin2[fam,1:T] <- a3_dz_twin2[fam,1:T]/sqrt(2)

        # Modelling phenotypes:
        data_dz_twin1[fam,1:T] ~ dmnorm(mu[1:T] + a2_dz[fam,1:T] +
                                        a4_dz_twin1[fam,1:T], tau_e[1:T,1:T])
        data_dz_twin2[fam,1:T] ~ dmnorm(mu[1:T] + a2_dz[fam,1:T] +
                                        a4_dz_twin2[fam,1:T], tau_e[1:T,1:T])
    }

    # Prior distributions

    # Normal prior for expected values of time points
    for (timepoint in 1:T){
```

```
43          mu[timepoint] ~ dnorm(0, .1)
44      }
45
46      # Inverse Wishart prior distribution for covariance matrices
47      tau_a[1:T,1:T] ~ dwish(omega_a[,], T+1)
48      tau_e[1:T,1:T] ~ dwish(omega_e[,], T+1)
49
50      # calculate inverse
51      Sigma_a[1:T,1:T] <- inverse(tau_a[,])
52      Sigma_e[1:T,1:T] <- inverse(tau_e[,])
53  }
```

Longitudinal ACE model

IRT approach (Latent trait model)

Following JAGS script fits a longitudinal ACE twin model with an incorporated IRT Rasch model at the phenotypic level (Latent trait ACE twin model).

```
#=============================================================================#
# Following JAGS script fits a longitudinal ACE latent trait-state AE twin model
# T = total number of time points (equal to 2 here)
# n_dz = total number of DZ twin families; n_dz = total number of MZ families
# n_items = total number of questionnaire items
#
# Note that this script works for 2 time points but can easily be adjusted
# to work for more time points.
# Any questions: Contact Inga Schwabe (I.Schwabe@uvt.nl)
#=============================================================================#

model{
    # Twin model part for MZ twins
    for (fam in 1:n_mz){

        a1_mz[fam,1:T] ~ dmnorm(mu_a[1:T], tau_a[1:T,1:T])
        c_mz[fam, 1:T] ~ dmnorm(mu_c[1:T], tau_c[1:T,1:T])

        # Modelling phenotypes:
        pheno_mz_twin1[fam,1:T] ~ dmnorm(mu[1:T] + a1_mz[fam,1:T] +
                                         c_mz[fam,1:T], tau_e[1:T,1:T])
        pheno_mz_twin2[fam,1:T] ~ dmnorm(mu[1:T] + a1_mz[fam,1:T] +
                                         c_mz[fam,1:T], tau_e[1:T,1:T])

        # IRT part: Rasch model for both time points
        for (k in 1:n_items){
            logit(p_mz_twin1_t1[fam,k]) <- pheno_mz_twin1[fam,1] - beta[k]
            data_mz_twin1_t1[fam,k] ~ dbern(p_mz_twin1_t1[fam,k])

            logit(p_mz_twin2_t1[fam,k]) <- pheno_mz_twin2[fam,1] - beta[k]
            data_mz_twin2_t1[fam,k] ~ dbern(p_mz_twin2_t1[fam,k])
        }

        for (k in 1:n_items){
            logit(p_mz_twin1_t2[fam,k]) <- pheno_mz_twin1[fam,2] - beta[k]
            data_mz_twin1_t2[fam,k] ~ dbern(p_mz_twin1_t2[fam,k])

            logit(p_mz_twin2_t2[fam,k]) <- pheno_mz_twin2[fam,2] - beta[k]
            data_mz_twin2_t2[fam,k] ~ dbern(p_mz_twin2_t2[fam,k])
        }
```

```
41      }
42
43      # Twin model part for DZ twins
44      for (fam in 1:n_dz){
45          a1_dz[fam,1:T] ~ dmnorm(mu_a[1:T], tau_a[1:T,1:T])
46          a2_dz[fam,1:T] <- a1_dz[fam,1:T]/sqrt(2)
47
48          a3_dz_twin1[fam,1:T] ~ dmnorm(mu_a[1:T], tau_a[1:T,1:T])
49          a3_dz_twin2[fam,1:T] ~ dmnorm(mu_a[1:T], tau_a[1:T,1:T])
50
51          a4_dz_twin1[fam,1:T] <- a3_dz_twin1[fam,1:T]/sqrt(2)
52          a4_dz_twin2[fam,1:T] <- a3_dz_twin2[fam,1:T]/sqrt(2)
53
54          c_dz[fam,1:T] ~ dmnorm(mu_c[1:T], tau_c[1:T,1:T])
55
56          # Modelling phenotypes:
57          pheno_dz_twin1[fam,1:T] ~ dmnorm(mu[1:T] + a2_dz[fam,1:T] +
58                                           a4_dz_twin1[fam,1:T] + c_dz[fam,1:T],
59                                           tau_e[1:T,1:T])
60          pheno_dz_twin2[fam,1:T] ~ dmnorm(mu[1:T] + a2_dz[fam,1:T] +
61                                           a4_dz_twin2[fam,1:T] + c_dz[fam,1:T],
62                                           tau_e[1:T,1:T])
63
64          # IRT part (Rasch model for both time points):
65          for (k in 1:n_items){
66              logit(p_dz_twin1_t1[fam,k]) <- pheno_dz_twin1[fam,1] - beta[k]
67              data_dz_twin1_t1[fam,k] ~ dbern(p_dz_twin1_t1[fam,k])
68
69              logit(p_dz_twin2_t1[fam,k]) <- pheno_dz_twin2[fam,1] - beta[k]
70              data_dz_twin2_t1[fam,k] ~ dbern(p_dz_twin2_t1[fam,k])
71          }
72
73          for (k in 1:n_items){
74              logit(p_dz_twin1_t2[fam,k]) <- pheno_dz_twin1[fam,2] - beta[k]
75              data_dz_twin1_t2[fam,k] ~ dbern(p_dz_twin1_t2[fam,k])
76
77              logit(p_dz_twin2_t2[fam,k]) <- pheno_dz_twin2[fam,2] - beta[k]
78              data_dz_twin2_t2[fam,k] ~ dbern(p_dz_twin2_t2[fam,k])
79          }
80      }
81
82      # Prior distributions
83      # Normal prior for beta item parameters:
84      for (k in 1:n_items){
85          beta[k] ~ dnorm(0, .1)
86      }
```

```
87
88      # The expected value of the first time point is set to 0 to identify the
89      # measurement scale. A normal prior is set on the remaining time point avera
90      mu[1] <- 0
91      mu[2] ~ dnorm(0, .1)
92
93      # Inverse Wishart prior distribution for covariance matrices
94      tau_a[1:T,1:T] ~ dwish(omega_a[,], T+1)
95      tau_e[1:T,1:T] ~ dwish(omega_e[,], T+1)
96      tau_c[1:T,1:T] ~ dwish(omega_c[,], T+1)
97
98      # Calculate inverse:
99      Sigma_a[1:T,1:T] <- inverse(tau_a[,])
100     Sigma_e[1:T,1:T] <- inverse(tau_e[,])
101     Sigma_c[1:T,1:T] <- inverse(tau_c[,])
102 }
```

IRT approach (Latent trait model): Alternative prior

Following JAGS script fits a longitudinal ACE twin model with an incorporated IRT Rasch model at the phenotypic level (Latent trait ACE twin model). This script was used in the additional simulation study.

```
#=============================================================================#
# Following JAGS script fits a longitudinal ACE latent trait-state AE twin model
# T = total number of time points (equal to 2 here)
# n_dz = total number of DZ twin families; n_dz = total number of MZ families
# n_items = total number of questionnaire items
#
# Note that this script works for 2 time points but can easily be adjusted
# to work for more time points.
# Any questions: Contact Inga Schwabe (I.Schwabe@uvt.nl)
#=============================================================================#

model{
    # Twin model part for MZ twins
    for (fam in 1:n_mz){

        a1_mz[fam,1:T] ~ dmnorm(mu_a[1:T], tau_a[1:T,1:T])
        c_mz[fam, 1:T] ~ dmnorm(mu_c[1:T], tau_c[1:T,1:T])

        # Modelling phenotypes:
        pheno_mz_twin1[fam,1:T] ~ dmnorm(mu[1:T] + a1_mz[fam,1:T] +
                                         c_mz[fam,1:T], tau_e[1:T,1:T])
        pheno_mz_twin2[fam,1:T] ~ dmnorm(mu[1:T] + a1_mz[fam,1:T] +
                                         c_mz[fam,1:T], tau_e[1:T,1:T])

        # IRT part: Rasch model for both time points
        for (k in 1:n_items){
            logit(p_mz_twin1_t1[fam,k]) <- pheno_mz_twin1[fam,1] - beta[k]
            data_mz_twin1_t1[fam,k] ~ dbern(p_mz_twin1_t1[fam,k])

            logit(p_mz_twin2_t1[fam,k]) <- pheno_mz_twin2[fam,1] - beta[k]
            data_mz_twin2_t1[fam,k] ~ dbern(p_mz_twin2_t1[fam,k])
        }

        for (k in 1:n_items){
            logit(p_mz_twin1_t2[fam,k]) <- pheno_mz_twin1[fam,2] - beta[k]
            data_mz_twin1_t2[fam,k] ~ dbern(p_mz_twin1_t2[fam,k])

            logit(p_mz_twin2_t2[fam,k]) <- pheno_mz_twin2[fam,2] - beta[k]
            data_mz_twin2_t2[fam,k] ~ dbern(p_mz_twin2_t2[fam,k])
        }
    }
```

```
42
43      # Twin model part for DZ twins
44      for (fam in 1:n_dz){
45          a1_dz[fam,1:T] ~ dmnorm(mu_a[1:T], tau_a[1:T,1:T])
46          a2_dz[fam,1:T] <- a1_dz[fam,1:T]/sqrt(2)
47
48          a3_dz_twin1[fam,1:T] ~ dmnorm(mu_a[1:T], tau_a[1:T,1:T])
49          a3_dz_twin2[fam,1:T] ~ dmnorm(mu_a[1:T], tau_a[1:T,1:T])
50
51          a4_dz_twin1[fam,1:T] <- a3_dz_twin1[fam,1:T]/sqrt(2)
52          a4_dz_twin2[fam,1:T] <- a3_dz_twin2[fam,1:T]/sqrt(2)
53
54          c_dz[fam,1:T] ~ dmnorm(mu_c[1:T], tau_c[1:T,1:T])
55
56          # Modelling phenotypes:
57          pheno_dz_twin1[fam,1:T] ~ dmnorm(mu[1:T] + a2_dz[fam,1:T] +
58                                           a4_dz_twin1[fam,1:T] + c_dz[fam,1:T],
59                                           tau_e[1:T,1:T])
60          pheno_dz_twin2[fam,1:T] ~ dmnorm(mu[1:T] + a2_dz[fam,1:T] +
61                                           a4_dz_twin2[fam,1:T] + c_dz[fam,1:T],
62                                           tau_e[1:T,1:T])
63
64          # IRT part (Rasch model for both time points):
65          for (k in 1:n_items){
66              logit(p_dz_twin1_t1[fam,k]) <- pheno_dz_twin1[fam,1] - beta[k]
67              data_dz_twin1_t1[fam,k] ~ dbern(p_dz_twin1_t1[fam,k])
68
69              logit(p_dz_twin2_t1[fam,k]) <- pheno_dz_twin2[fam,1] - beta[k]
70              data_dz_twin2_t1[fam,k] ~ dbern(p_dz_twin2_t1[fam,k])
71          }
72
73          for (k in 1:n_items){
74              logit(p_dz_twin1_t2[fam,k]) <- pheno_dz_twin1[fam,2] - beta[k]
75              data_dz_twin1_t2[fam,k] ~ dbern(p_dz_twin1_t2[fam,k])
76
77              logit(p_dz_twin2_t2[fam,k]) <- pheno_dz_twin2[fam,2] - beta[k]
78              data_dz_twin2_t2[fam,k] ~ dbern(p_dz_twin2_t2[fam,k])
79          }
80      }
81
82      # Prior distributions
83      # Normal prior for beta item parameters:
84      for (k in 1:n_items){
85          beta[k] ~ dnorm(0, .1)
86      }
87
```

```
88      # The expected value of the first time point is set to 0 to identify the
89      # measurement scale.
90      # A normal prior is set on the remaining time point averages
91      mu[1] <- 0
92      mu[2] ~ dnorm(0, .1)
93
94      # Inverse Wishart prior distribution for covariance matrices
95      tau_a[1:T,1:T] ~ dwish(omega_a, nu_a)
96      tau_c[1:T,1:T] ~ dwish(omega_c, nu_c)
97      tau_e[1:T,1:T] ~ dwish(omega_e, nu_e)
98
99      # Calculate inverse:
100     Sigma_a[1:T,1:T] <- inverse(tau_a[,])
101     Sigma_e[1:T,1:T] <- inverse(tau_e[,])
102     Sigma_c[1:T,1:T] <- inverse(tau_c[,])
103
104     # Hyperprios for the inverse Wishart priors
105
106     # Hyperpriors covariance matrix
107     for (d in 1:2){
108         omega_a[d,d] <- lambda_a[d]
109         omega_c[d,d] <- lambda_c[d]
110         omega_e[d,d] <- lambda_e[d]
111
112         lambda_a[d] <- exp(Loglambda_a[d])
113         lambda_c[d] <- exp(Loglambda_c[d])
114         lambda_e[d] <- exp(Loglambda_e[d])
115
116         Loglambda_a[d] ~ dunif(-100,100)
117         Loglambda_c[d] ~ dunif(-100,100)
118         Loglambda_e[d] ~ dunif(-100,100)
119     }
120
121     # Hyperiors degree of freedom
122     # 2 = total number of timepoints
123     nu_a ~ dunif(2,100)
124     nu_c ~ dunif(2,100)
125     nu_e ~ dunif(2,100)
126
127     # Set off diag elements to 0
128     omega_a[1,2] <- 0
129     omega_a[2,1] <- 0
130
131     omega_c[1,2] <- 0
132     omega_c[2,1] <- 0
133
```

```
134         omega_e[1,2] <- 0
135         omega_e[2,1] <- 0
136   }
```
